# Supplementary material for: Content-rich biological network constructed by mining PubMed abstracts
Source: BMC Bioinformatics. 2004 Oct 8;5:147. doi: 10.1186/1471-2105-5-147 (PMC528731; doi:10.1186/1471-2105-5-147)
Supplement: Additional File 2 — The original results of the above study (non-essential files are deleted to keep the file size under the limit set by BMC bioinformatics). [file 1471-2105-5-147-S2.bz2 › chilibotAdditionalFile2/dip05/65ID7729427E225/html/TAF12_TBP.html]

 


 **TAF12** and **TBP** 
  
Found 58 abstracts in PubMed, retrieved 05.  
 

 What does Google say? 
 PDF only 
| .edu only 

---

**Interactive relationship** (e.g. stimulation, inhibition, etc)

**Neutral relationship**- Finally, our results suggest that  **TBP**  associated factor  [ **TAF12** ]  components of SAGA are differentially required for  **TBP**  binding to SAGA dependent promoters.  Ref: 12370284 Mol Cell Biol, 2002
- The MADF domain directs sequence specific DNA binding to a site consisting of multiple trinucleotide repeats, while the BESS domain directs a variety of protein protein interactions, including interactions with itself, with Dorsal, and with a  **TBP**  associated factor  [ **TAF12** ] .  Ref: 12459265 Gene, 2002

**Non-interactive relationship** (e.g. studied together, co-existance, homology, etc.)

- Of these 400, interferon regulatory factor 4 IRF4, cyclin B2,  **TBP**  associated factor  [ **TAF12** ] , eukaryotic elongation factor and pim 2 were up regulated more than 3.5 fold.  Ref: 11790884 Dis Markers, 2001
- Interestingly, a set of genes affected in the taf1 DeltaTAND mutant is similarly affected in the  **taf12**  HFD mutants but not in the nsl mutants of  **TBP** .  Ref: 12582246 Nucleic Acids Res, 2003
- Mutations in the histone fold domain of the  **TAF12**  gene show synthetic lethality with the TAF1 gene lacking the TAF N terminal domain TAND by different mechanisms from those in the SPT15 gene encoding the TATA box binding protein  [ **TBP** ]   **TBP** .  Ref: 12582246 Nucleic Acids Res, 2003
